# Experimental malaria-associated acute kidney injury is independent of parasite sequestration and resolves upon antimalarial treatment

**DOI:** 10.3389/fcimb.2022.915792

**Published:** 2022-08-08

**Authors:** Hendrik Possemiers, Emilie Pollenus, Fran Prenen, Sofie Knoops, Priyanka Koshy, Philippe E. Van den Steen

**Affiliations:** ^1^ Laboratory of Immunoparasitology, Department of Microbiology, Immunology and Transplantation, Rega Institute for Medical Research, KU Leuven, KU, Leuven, Belgium; ^2^ Department of Pathology, University Hospitals Leuven, Leuven, Belgium

**Keywords:** malaria, kidney, sequestration, resolution, inflammation

## Abstract

Malaria remains a important global disease with more than 200 million cases and 600 000 deaths each year. Malaria-associated acute kidney injury (MAKI) may occur in up to 40% of patients with severe malaria and is associated with increased mortality. Histopathological characteristics of AKI in malaria are acute tubular injury, interstitial nephritis, focal segmental glomerulosclerosis, collapsing glomerulopathy and glomerulonephritis. We observed that C57BL/6 mice infected with *Plasmodium berghei* NK65 (*Pb*NK65) develop MAKI in parallel with malaria-associated acute respiratory distress syndrome (MA-ARDS). MAKI pathology was associated with proteinuria, acute tubular injury and collapse of glomerular capillary tufts, which resolved rapidly after treatment with antimalarial drugs. Importantly, parasite sequestration was not detected in the kidneys in this model. Furthermore, with the use of skeleton binding protein-1 (SBP-1) KO *Pb*NK65 parasites, we found that parasite sequestration in other organs and its subsequent high parasite load are not required for the development of experimental MAKI. Similar proteinuria, histopathological features, and increases in kidney expression of interferon-γ, TNF-α, kidney injury molecule-1 (KIM-1) and heme oxygenase-1 (HO-1) was observed in both infected groups despite a significant difference in parasite load. Taken together, we introduce a model of experimental AKI in malaria with important similarities to AKI in malaria patients. Therefore, this mouse model might be important to further study the pathogenesis of AKI in malaria.

## Introduction

Malaria remains a important global disease with more than 200 million clinical cases and 600 000 deaths each year (World Health Organization, 2021). Five different *Plasmodium* species are known to infect humans, with *Plasmodium falciparum* and *Plasmodium vivax* causing most of the infections. Malaria-associated acute kidney injury (MAKI) is a common complication in severe malaria, affecting up to 40% of the adults patients (Koopmans et al., 2015; Conroy et al., 2016). Recent studies also showed the presence of MAKI in 45 to 60% of children with severe malaria (Conroy et al., 2016; Oshomah-Bello et al., 2020). MAKI is associated with increased mortality, which led to case fatality rates up to 75% without appropriate renal replacement therapy (Trang et al., 1992; Conroy et al., 2016). The most important histopathological characteristics of AKI in malaria are acute tubular injury, interstitial nephritis, focal segmental glomerulosclerosis, collapsing glomerulopathy and glomerulonephritis (Nguansangiam et al., 2007; Mishra and Das, 2008; Amoura et al., 2020).

To investigate the pathogenesis of AKI in malaria, several malaria mouse models of AKI have been used in the past. AKI has been described in C57BL/6 mice infected with *Plasmodium berghei* ANKA (*Pb*ANKA) in various studies in parallel with experimental cerebral malaria (ECM) (Abreu et al., 2014; Souza et al., 2015; Silva et al., 2018). AKI pathology in this model was characterized by an increase in plasma concentrations of blood urea nitrogen (BUN) and creatinine, proteinuria, glomerular hypercellularity and increased expression of pro-inflammatory cytokines in the kidney. Similar MAKI pathology was described in BALB/c mice infected with *Pb*ANKA (Elias et al., 2012). In addition, Yashima et al. observed MAKI in NC mice infected with *P. chabaudi* AS (Yashima et al., 2017). This was characterized by mesangial proliferative glomerulonephritis with endothelial damage, proteinuria and the deposition of immunoglobulin G and complement component 3 in mesangium and glomerular capillaries.

Parasite sequestration, which is the binding of infected red blood cells (iRBCs) to endothelial cells (ECs), has been proposed as a potential contributor to AKI pathogenesis in malaria (Katsoulis et al., 2021). Post-mortem studies observed parasite sequestration of *P. falciparum* in glomerular and peritubular capillaries in adults and children with MAKI (Nguansangiam et al., 2007; Milner et al., 2014). Sequestration is mediated by specific parasite adhesins, which are expressed on the surface of the iRBCs. The export of the parasite adhesins to the surface of the iRBCs is mediated by several parasite proteins, including skeleton binding protein-1 (SBP-1) in the Maurer’s cleft. SBP-1 is both expressed in human and rodent malaria parasites (De Niz et al., 2016). The export system for the adhesins is relatively conserved but the adhesins mediating sequestration of *P. falciparum* and *P. berghei* are not conserved. The *Plasmodium falciparum* erythrocyte membrane protein-1 (*Pf*EMP-1) adhesins encoded by the *var* genes mediate sequestration of *P. falciparum* parasites (Scherf et al., 2008). These *var* genes are absent in the genome of *P. berghei* and the adhesins mediating sequestration of *P. berghei* are currently not known (Possemiers et al., 2021). De Niz et al. showed that knock-out (KO) of SBP-1 in *Pb*ANKA led to reduced sequestration, the absence of the development of ECM and also a lower parasitemia, consistent with the notion that sequestering parasites avoid splenic clearance (De Niz et al., 2016). Moreover, we showed that SBP-1 KO-mediated parasite sequestration had no influence on the development of experimental MA-ARDS but inhibited the spontaneous resolution of MA-ARDS (Possemiers et al., 2021).

A recent study by Amoura et al. found collapsing focal segmental glomerulosclerosis (FSGS) in 91% of kidney biopsies from malaria patients after successful treatment of malaria (Amoura et al., 2020). In addition, various *P. falciparum* cases with collapsing FSGS were reported (Niang et al., 2008; Kute et al., 2013; Sehar et al., 2015). This shows that malaria may be a new causal factor for secondary FSGS. FSGS is the most common primary glomerular histologic lesion associated with proteinuria and with end-stage renal disease (Bose and Cattran, 2014). The primary form of FSGS is idiopathic and the secondary form includes genetic, virus-associated and drug-induced etiologies (D’Agati et al., 2004; Rosenberg and Kopp, 2017).

In this study, we observed that C57BL/6 mice infected with *Pb*NK65 Edinburgh strain (*Pb*NK65-E) parasites develop AKI in parallel with malaria-associated acute respiratory distress syndrome (MA-ARDS). AKI pathology was associated with increased proteinuria, acute tubular injury and collapse of glomerular capillary tufts, which resolved rapidly after treatment with antimalarial drugs. Furthermore, we investigated the role of sequestration in experimental AKI by using SBP-1 KO *Pb*NK65 parasites, which have a decreased capacity to sequester, in compared to the wildtype (WT) *Pb*NK65 parasites. We did not observe direct parasite sequestration in the kidneys but parasite load was significantly reduced in SBP-1 KO infected mice compared to WT infected mice. However, both infected groups had similar renal histopathological features, similar increased renal expression of interferon-γ (IFN-γ), TNF-α, kidney injury molecule-1 (KIM-1) and heme oxygenase-1 (HO-1) and similar increased proteinuria. These data suggest that parasite sequestration and subsequent high parasite load are not required for the development of experimental AKI.

## Materials and methods

### Ethical statement

All experiments at the KU Leuven were performed according to the regulations of the European Union (directive 2010/63/EU) and the Belgian Royal Decree of 29 May 2013, and were approved by the Animal Ethics Committee of the KU Leuven (License LA1210251, project P196/2015 and license LA1210186, project P052/2020, Belgium).

### Parasites and mice

Equal numbers of male and female C57BL/6 mice were obtained from Janvier (7–8 weeks old, Le Genest-Saint-Isle, France). All mice were housed in individually ventilated cages in a 12 h light and 12 h dark cycle in SPF animal facility. Drinking water was supplemented with 4-amino benzoic acid (0.422 mg/ml PABA, Sigma-Aldrich, Bornem, Belgium).

For ART + CQ treatment experiments, C57BL/6 mice were infected with *P. berghei* NK65 Edinburgh strain (*Pb*NK65-E) parasites by intraperitoneal (i.p.) injection of 10^4^ iRBCs as described previously (Van den Steen et al., 2010). For WT vs. SBP-1 KO experiments, C57BL/6 mice were infected with *P. berghei* NK65 2168cl2 (WT) (Pham et al., 2017) or SBP-1 KO *P. berghei* NK65 2559cl2 (SBP-1 KO) (Possemiers et al., 2021) parasites by i.p. injection of 10^4^ iRBCs.

### Scoring of disease progression and parasitemia determination

Parasitemia, body weight and clinical score were evaluated daily starting from day 5 post infection (p.i.) in the WT vs. SBP-1 KO experiments and from day 6 p.i. in ART + CQ treatment experiments. Blood smears of tail blood were stained with 10% Giemsa (VWR, Heverlee, Belgium) and parasitemia was calculated by microscopic analysis. The clinical score was calculated by evaluating different clinical parameters including social activity (SA), limb grasping (LG), body tone (BT), trunk curl (TC), pilo-erection (PE), shivering (Sh), abnormal breathing (AB), dehydration (D), incontinence (I) and paralysis (P). A score of 0 (absent) or 1 (present) was given for TC, PE, Sh and AB and 0 (normal), 1 (intermediate) or 2 (most serious) for the other parameters. The total clinical score was calculated using the following formula: SA + LG + BT + TC + PE + 3 * (Sh + AB + D + I + P). In WT vs. SBP-1 KO experiments and ART + CQ treatment experiments mice were euthanized when the body weight decrease was >20% and >25% respectively compared to day 0 p.i. or when clinical score reached 10 or more.

### Antimalarial treatment

Where indicated, mice were treated with antimalarial drugs from day 8 p.i. to day 12 p.i. with a combination of artesunate (ART, 10 mg/kg in 0.9% NaCl with 0.1% NaHCO_3_; Sigma-Aldrich) and chloroquine diphosphate salt (CQ, 30 mg/kg in 0.9% NaCl; Sigma-Aldrich) *via* a daily i.p. injection of 100 µl as described by Pollenus et al. (Pollenus et al., 2021).

### Retro-orbital puncture and dissection

In part of the WT vs. SBP-1 KO experiments, mice were anesthetized with 3% isoflurane (Iso-Vet, Dechra, Nortwhich, United Kingdom) before retro-orbital puncture was performed with a heparinized (LEO Pharma, Lier, Belgium) glass capillary tube (Hirschmann-Laborgeräte, Eberstadt, Germany). The collected blood was injected in a cartridge in the Epoc Blood Analysis System (Siemens, Munich, Germany) for biochemical analysis. After the blood collection, mice were euthanized by performing heart puncture under anaesthesia with 3% isoflurane. After a transcardial perfusion with 20 ml PBS the left kidney was removed, laterally cut into two equal pieces and fixed in 4% formaldehyde (Klinipath, Duiven, The Netherlands) for 48 h at 4°C.

In the ART + CQ treatment experiments and part of the WT vs. SBP-1 KO experiments, mice were euthanized with Dolethal (Vétoquinol, Aartselaar, Belgium; 200 mg/mL, i.p. injection of 100 µL) followed by heart puncture at indicated time points. After a transcardial perfusion with 20 ml PBS the left kidney was removed and laterally cut into two equal pieces and fixed in 4% formaldehyde for 48 h at 4°C. In the plasma samples obtained from heart puncture, BUN levels were measured with the Urea Nitrogen (BUN) Colorimetric Detection Kit (Thermo Fisher Scientific Inc., Waltham, MA, USA) for the WT vs. SBP-1 KO experiments.

### Analysis of urine samples

Urine samples were collected in a 1.5 mL Eppendorf tube at the indicated time points in the morning. The albumin/creatinine ratio in the urine was determined to assess proteinuria and kidney function as described in Vandermosten et al. (Vandermosten et al., 2018).

### Kidney histology

After fixation, kidney tissues were dehydrated by applying gradually increasing ethanol concentrations in the Excelsior MS tissue processor (Thermo Fisher Scientific, Waltham, USA). Next, the tissues were embedded in paraffin with the HistoStar Workstation (Thermo Fisher Scientific) and 5 µm thick tissue sections were made using with Microm HM 355S microtome (Thermo Fisher Scientific). Tissue sections were stained with the Periodic Acid Schiff’s (PAS) staining kit (Carl Roth GmbH, Karlsruhe, Germany). Histological assessment was performed with a Leica DM 2000 microscope. The percentage of collapsed glomeruli and renal blood vessels with intravascular accumulation of one or more leukocytes was calculated on whole sections.

### Quantitative reverse transcription-polymerase chain reaction (qRT-PCR)

RNeasy Mini Kit (Qiagen, Hilden, Germany) was used to extract RNA from the kidney after mechanical homogenisation in RLT buffer. After extraction, RNA was quantified and cDNA was synthesized using the High Capacity cDNA Reverse Transcription Kit (Applied Biosystems, Waltham, USA). ABI Prism 7500 Sequence Detection System (Applied Biosystems) was used to perform qRT-PCR reaction on cDNA with specific primers (IDT, Leuven, Belgium, **Table 1**) in the TaqMan^®^ Fast Universal PCR master mix (Applied Biosystems). The relative mRNA expression was determined as 2^-ΔΔCT^, normalized to the mean 2^-CT^ value of the uninfected control mice and to the 2^-CT^ value of the 18S housekeeping gene.

**Table 1:** List of primers used for RT-qPCR.

Predesigned qPCR assays (IDT)			
Name	Ref Seq	Exon location	Assay ID
18S	NR_003286(1)	Exon 1-1	Hs.PT.39a.22214856.g
HO-1	NM_010442(1)	Exon 3-4	Mm.PT.51.8600055
IFN-γ	NM_008337(1)	Exon 1-2	Mm.PT.58.41769240
KIM-1	NM_001166631(3)	Exon 3-4	Mm.PT.58.43283412
TNF-α	NM_013693(1)	Exon 2-4	Mm.PT.58.12575861

### Statistical analysis

Statistical analysis was done using the GraphPad Prism software (GraphPad software, San Diego, USA, version 8.3.1). The non-parametric Mann–Whitney U test was used to determine the statistical significance between two groups. P-values smaller than 0.05 were considered statistically significant. P-values were defined as follows: *p < 0.05, **p < 0.01, ***p < 0.001, ****p < 0.0001. To correct for multiple testing, the Holm-Bonferroni method was applied when 4 or more comparisons were made. Unless otherwise specified, each dot represents the result from an individual mouse. Horizontal lines represent group medians. Asterisks without horizontal lines represent significant differences compared to the control group. Horizontal lines with asterisk on top indicate significant differences between groups.

## Results

### Malaria-associated acute kidney injury occurs in C57BL/6 mice infected with *Pb*NK65-E parasites and resolves upon antimalarial treatment

**Figure 1:**
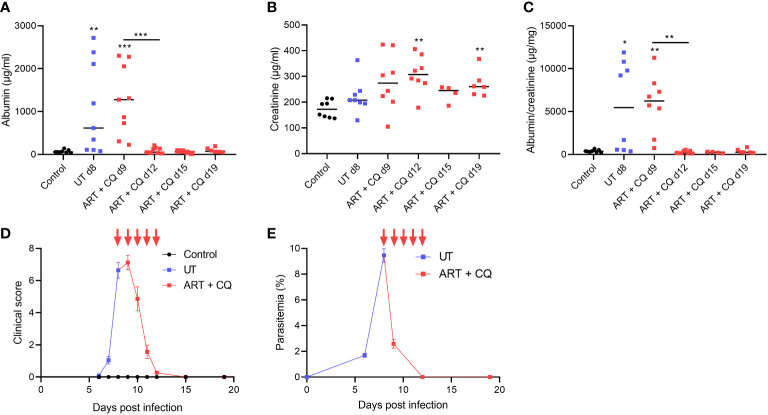
Antimalarial treatment decreased proteinuria in *Pb*NK65-E infected mice. C57BL/6 mice were infected with *Pb*NK65-E and were treated daily from day 8 until day 12 p.i. with 10 mg/kg ART + 30 mg/kg CQ (ART+CQ). **(A–C)** Albumin, creatinine and albumin/creatinine ratios in the urine were determined. **(D)** The clinical score was monitored daily from day 6 p.i. onwards, daily ART + CQ treatment indicated with red arrows. **(E)** Peripheral parasitemia was determined on blood smears, daily ART + CQ treatment indicated with red arrows. Asterisks above data points indicate significant differences compared to control mice, asterisks above a horizontal line show significant differences between different time points, *p < 0.05, **p < 0.01, ***p < 0.001. **(A–C)** Data of two experiments, Control: n = 8, untreated *Pb*NK65-E infected mice (UT) d8: n = 8-9, ART+CQ d9-d19: n = 4-9. **(D, E)** Data of two experiments, Control: n = 6, UT/ART+CQ: n = 6-22. Mann-Whitney U test with Holm-Bonferroni correction for multiple testing (number of tests = 6) was performed.

We observed that C57BL/6 mice infected with *Pb*NK65-E, a model for experimental MA-ARDS, develop MAKI in parallel with lung pathology. At day 8 post infection (p.i.), proteinuria was observed with a significant increase in albumin/creatinine ratio in the *Pb*NK65-E infected mice compared to the control mice (**Figures 1A–C**). Furthermore, we studied the effect of antimalarial treatment on MAKI. *Pb*NK65-E infected mice were treated with artesunate and chloroquine (ART + CQ) from day 8 until day 12 p.i. as described by Pollenus et al. (Pollenus et al., 2021). In this model, resolution of MA-ARDS occurs from day 8 p.i. to day 15 p.i., with alveolar edema and clinical score resolving to control level by day 15 p.i (Pollenus et al., 2021). A significant decrease in proteinuria was observed in the treated mice from day 9 p.i. to day 12 p.i. (**Figures 1A–C** and **Supplementary Figure 1**). This decrease occurred in parallel with a decrease in clinical score and parasitemia (**Figures 1D, E**).

**Figure 2:**
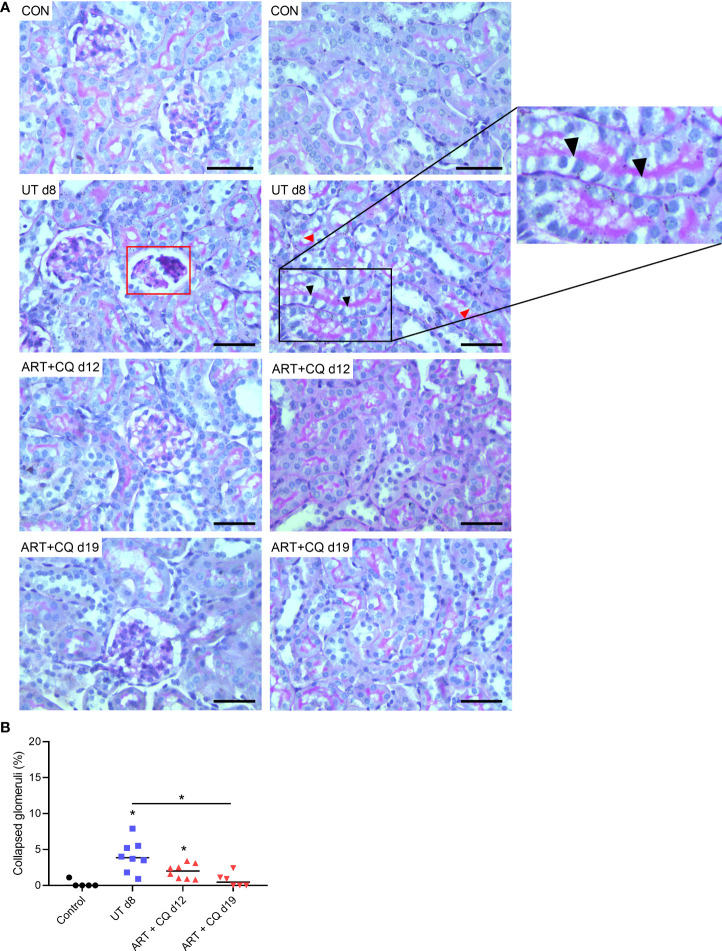
Antimalarial treatment decreased tubular injury and collapse of glomerular capillary tuft in kidneys of *Pb*NK65-E infected mice. C57BL/6 mice were infected with *Pb*NK65-E parasites and kidneys were dissected at the indicated times. From day 8 until day 12 p.i., mice were treated daily with antimalarial drugs (ART + CQ). **(A)** Sections were stained with PAS. Representative images are shown (original magnification x40, bar = 50 µm). Red frames indicate collapse of glomerular tufts. Black arrowheads indicate vacuolization of proximal tubular epithelial cells, red arrowheads indicate loss of brush border of proximal tubular epithelial cells. **(B)** Percentage of glomeruli with collapsed glomerular tufts counted on PAS stained kidney sections Asterisks above data points indicate significant differences compared to control mice, asterisks above a horizontal line show significant differences between different time points, *p < 0.05. Data of two experiments, Control: n = 6, untreated *Pb*NK65-E infected mice (UT) d8: n = 8, ART+CQ d12: n = 8, ART+CQ d19: n = 6. Mann-Whitney U test with Holm-Bonferroni correction for multiple testing (number of tests = 6) was performed in panel **(B)**.

We performed Periodic Acid Schiff (PAS) staining on kidney sections of control and *Pb*NK65-E infected mice with and without antimalarial treatment to study the histopathology of experimental MAKI. At day 8 p.i., acute tubular injury was observed with vacuolization and occasional loss of brush border of proximal tubular epithelial cells, which was more pronounced in the cortex of the kidney (**Figure 2A**). Sclerosis of the glomeruli was also detected, with the collapse of glomerular capillary tufts in <10% of glomeruli (**Figures 2A, B** and **Supplementary Figure 2**).

**Figure 3:**
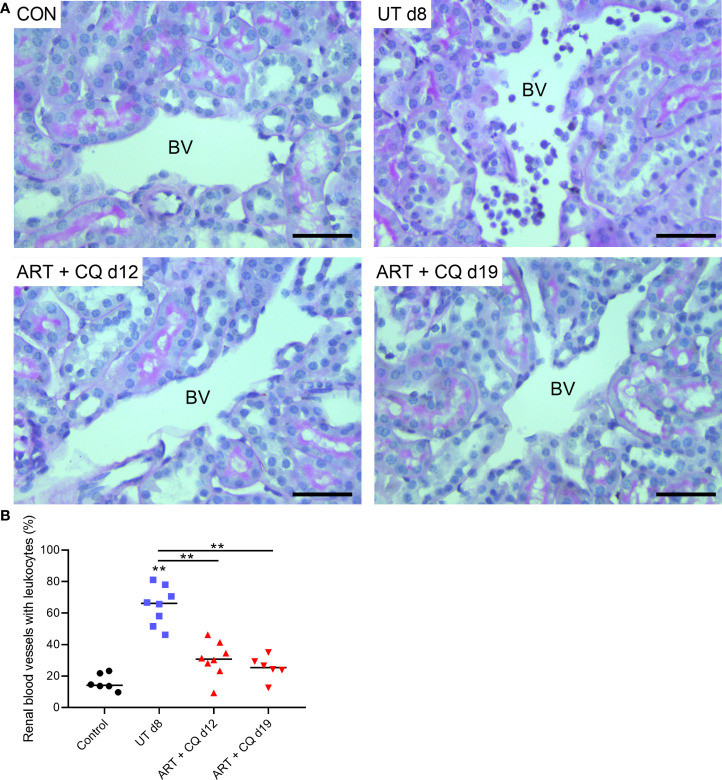
Antimalarial treatment decreased intravascular accumulation of leukocytes in kidneys of *Pb*NK65-E infected mice. C57BL/6 mice were infected with *Pb*NK65-E parasites and at day 8 p.i. kidneys were dissected. C57BL/6 mice infected with *Pb*NK65-E were treated from day 8 to day 12 p.i. with antimalarial drugs (ART + CQ) and at day 12 and 19 p.i. mice were dissected. Kidney sections were stained with PAS. **(A)** Representative images are shown (original magnification x40, bar = 50 µm). BV indicates blood vessel on renal section. **(B)** Percentage of renal blood vessels with intravascular accumulation of leukocytes Asterisks above data points indicate significant differences compared to control mice, asterisks above a horizontal line show significant differences between different time points, **p < 0.01. Data of two experiments, Control: n = 6, untreated *Pb*NK65-E infected mice (UT) d8: n = 8, ART+CQ d12: n = 8, ART+CQ d19: n = 6.

The kidneys of the ART + CQ treated mice showed resolution of the tubular damage in the cortical region with almost no vacuolization and normal brush border of the proximal tubular epithelial cells at day 12 and 19 p.i. (**Figure 2A**). Moreover, a significant decrease in percentage of collapsed glomerular capillary tufts in the kidneys of the ART + CQ treated mice was observed from day 8 p.i. to day 19 p.i. (**Figure 2B**). Moreover, increased intravascular accumulation of leukocytes was detected in kidneys of *Pb*NK65-E infected mice at day 8 p.i. compared to control mice or ART + CQ treated mice (**Figures 3A, B**).

Overall, these data suggest that infection of C57BL/6 mice with *Pb*NK65 leads to mild but significant MAKI and that the MAKI pathology is resolved by antimalarial treatment.

### SBP-1-mediated sequestration and subsequent high parasite load is dispensable for the development of MAKI

To study the role of whole-body parasite sequestration and subsequent high parasite load on the pathogenesis of experimental AKI, C57BL/6 mice were infected with transgenic WT and SBP-1 KO *Pb*NK65 parasites and the AKI pathology was compared. We previously showed with bioluminescence imaging and RT-qPCR that the SBP-1 KO *Pb*NK65 has a significantly reduced sequestration capacity compared to WT parasites with subsequent reduced parasite load and parasitemia (Possemiers et al., 2021).

The WT clone, which is used in the WT versus SBP-1 KO experiments, was generated from the *Pb*NK65-E line and is characterized by a slightly faster growth than the original *Pb*NK65-E line (Pham et al., 2017). Therefore, parasitemia and pathological symptoms occur 1 day earlier than with the original *Pb*NK65-E line in the above-described experiments. Otherwise, the pathology and disease course are similar. The SBP-1 KO parasites were generated from the WT clone and the effects on disease course and lung pathology were described previously (Possemiers et al., 2021).

**Figure 4:**
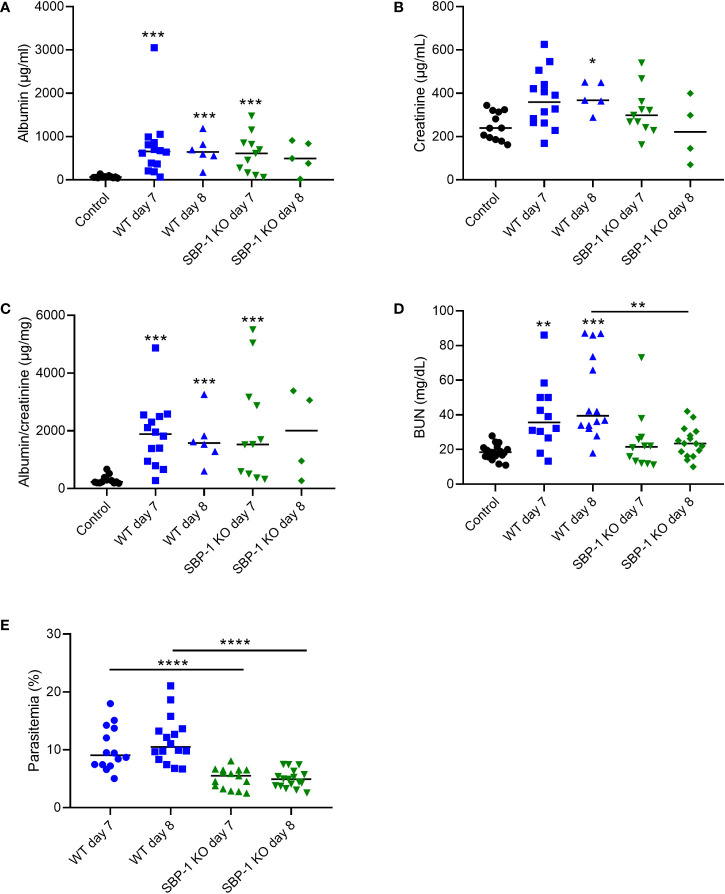
Similar proteinuria in WT and SBP-1 KO infected mice. C57BL/6 mice were infected with WT and SBP-1 KO *Pb*NK65 parasites. **(A–C)** Albumin, creatinine and albumin/creatinine ratios were determined in urine samples. **(D)** BUN values were determined in blood collected *via* retro-orbital puncture or in plasma collected *via* heart puncture. **(E)** Peripheral parasitemia was determined on blood smears. Asterisks above data points indicate significant differences compared to control mice, asterisks above a horizontal line show significant differences between infected groups, *p < 0.05, **p < 0.01, ***p < 0.001, ****p < 0.0001. **(A–C)** Data of 4 experiments, Control: n = 12, WT d7-d8: n = 5-14, SBP-1 KO d7-d8: n = 4-11, **(D, E)** data of 8 experiments, Control: n = 18, WT d7-d8: n = 12-16, SBP-1 KO d7-d8: n = 12-17. Mann-Whitney U test with Holm-Bonferroni correction for multiple testing (number of tests = 6) was performed.

Proteinuria was similarly increased in both WT and SBP-1 KO infected mice at day 7 and 8 p.i., when disease symptoms are at their peak. (**Figures 4A–C**). Parasitemia in the SBP-1 KO infected mice was significantly lower compared to the WT infected mice at day 7 and 8 p.i. (**Figure 4E**). This suggests that SBP-1 mediated sequestration and parasite load has no influence on proteinuria, as measured by the urinary albumin/creatinine ratio. BUN values were significantly increased in the WT infected mice at day 7 and day 8 p.i. compared to the control mice (**Figure 4D**). Despite similar proteinuria, a significant difference was observed in the BUN values between WT and SBP-1 KO infected mice at 8 days p.i., which might be related to extrarenal causes (**Figure 4D**).

**Figure 5:**
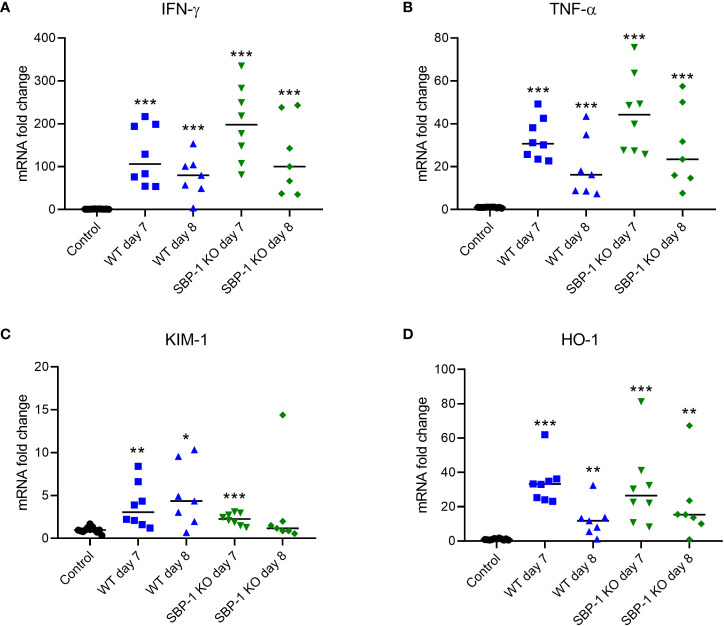
Increased expression of inflammatory cytokines, kidney injury molecule-1 and heme oxygenase 1 in kidneys of WT and SBP-1 KO infected mice. C57BL/6 mice were infected with WT and SBP-1 KO *Pb*NK65 parasites and at day 7 and 8 p.i. kidneys were dissected. Kidneys mRNA levels of **(A)** IFN-γ, **(B)** TNF-α, **(C)** KIM-1 and **(D)** HO-1 were measured by qRT-PCR. Asterisks above data points indicate significant differences compared to control mice, asterisks above a horizontal line show significant differences between infected groups ,*p < 0.05, **p < 0.01, ***p < 0.001. Data of 3 experiments, Control: n = 12, WT d7-d8: n = 7-8, SBP-1 KO d7-d8: n = 7-8. Mann-Whitney U test with Holm-Bonferroni correction for multiple testing (number of tests = 6) was performed.

AKI pathogenesis is associated with immune cell infiltration and increased cytokine secretion in the renal tissue (Zuk and Bonventre, 2016). Therefore, we measured the expression of inflammatory cytokines in the kidney at mRNA level. The mRNA expression of the inflammatory cytokines IFN-γ and TNF-α was significantly increased in the kidneys of both WT and SBP-1 KO infected mice at day 7 and 8 p.i. compared to the control group (**Figures 5A, B**).

The mRNA expression of KIM-1, a marker of early AKI, was also significantly increased at day 7 and day 8 p.i. in the WT infected mice and at day 7 p.i. in the SBP-1 KO infected mice (**Figure 5C**). The increase in renal KIM-1 expression was similar in WT and SBP-1 KO infected mice, further suggesting a similar AKI pathology in both infected groups.

The renal mRNA expression of HO-1 was measured, which has anti-inflammatory effects and is protective against kidney injury (Courtney and Maxwell, 2008; Srivastava et al., 2020). We observed in both WT and SBP-1 KO infected mice a significant increase in the renal expression of HO-1 compared to the control group (**Figure 5D**).

### Tubular injury and collapse of glomerular capillary tuft in kidneys of C57BL/6 mice infected with WT and SBP-1 KO *Pb*NK65

**Figure 6:**
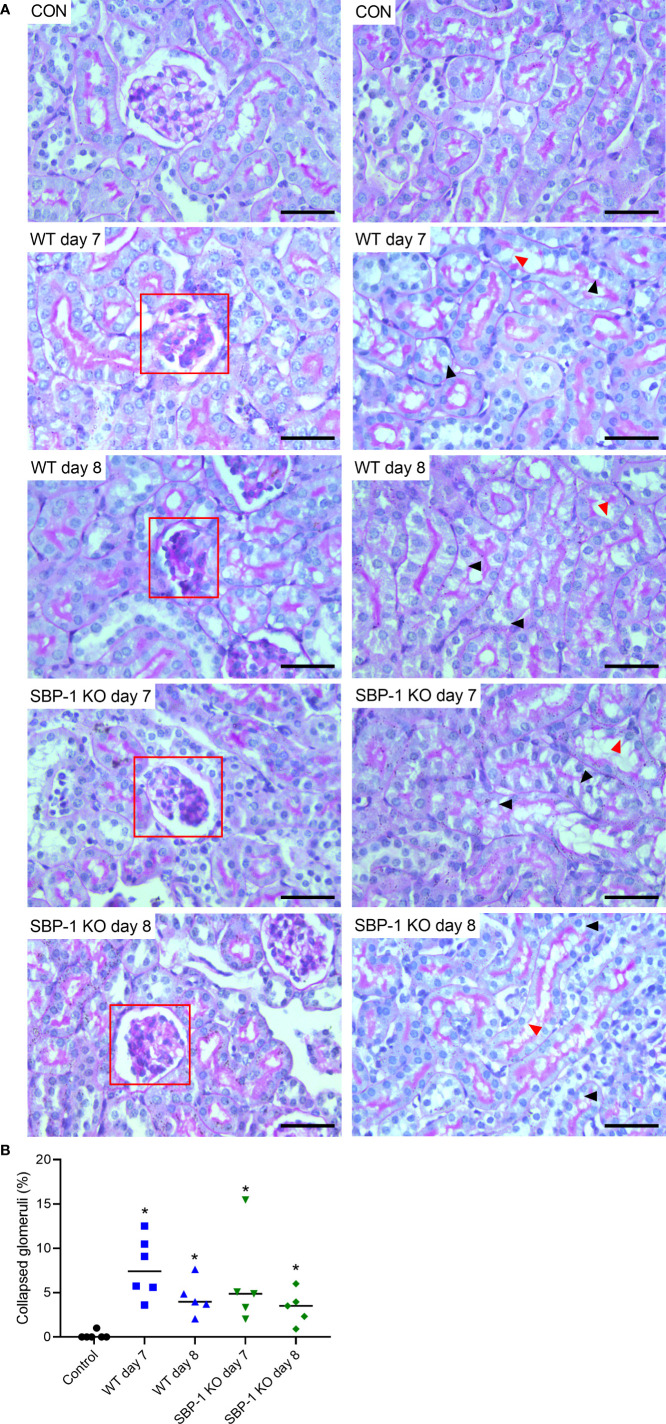
Similar tubular injury and collapse of glomerular capillary tuft in kidneys of WT and SBP-1 KO infected mice. C57BL/6 mice were infected with WT and SBP-1 KO *Pb*NK65 parasites and at day 7 and 8 p.i. kidneys were dissected. **(A)** Sections were stained with PAS. Representative images are shown (original magnification x40, bar = 50 µm). Red frames indicate collapse of glomerular tufts. Black arrowheads indicate vacuolization of proximal tubular epithelial cells, red arrowheads indicate loss of brush border of proximal tubular epithelial cells. **(B)** Percentage of glomeruli with collapsed glomerular tufts counted on PAS stained kidney sections Asterisks above data points indicate significant differences compared to control mice, *p < 0.05. Data of two experiments, Control: n = 6, WT d7-d8: n = 5-6, SBP-1 KO d7-d8: n = 5. Mann-Whitney U test with Holm-Bonferroni correction for multiple testing (number of tests = 6) was performed in panel B.

**Figure 7:**
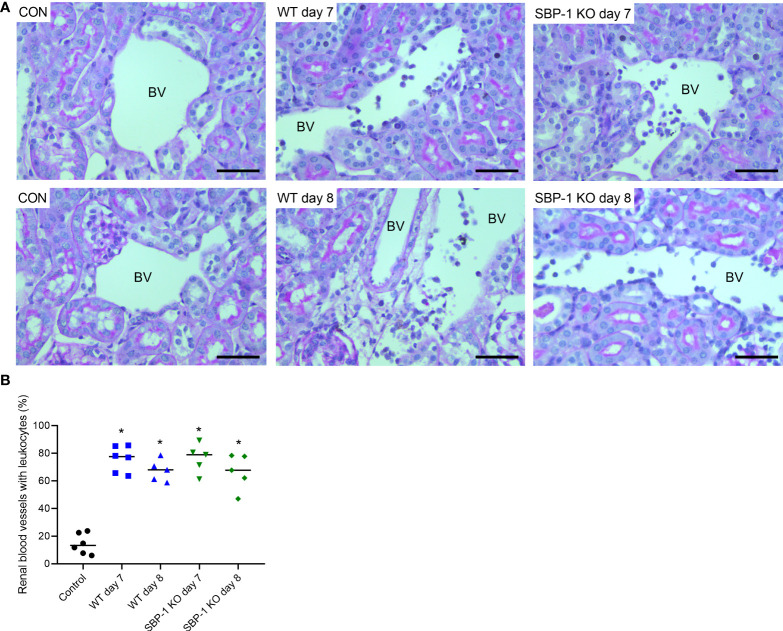
Similar intravascular accumulation of leukocytes in the kidneys of WT and SBP-1 KO infected mice. C57BL/6 mice were infected with WT and SBP-1 KO *Pb*NK65 parasites and at day 7 and 8 p.i. kidneys were dissected. Kidney sections were stained with PAS. **(A)** Representative images are shown (original magnification x40, bar = 50 µm). BV indicates blood vessel on renal section. **(B)** Percentage of renal blood vessels with intravascular accumulation of leukocytes Asterisks above data points indicate significant differences compared to control mice, *p < 0.05. Data of two experiments, Control: n = 6, WT d7-d8: n = 5-6, SBP-1 KO d7-d8: n = 5. Mann-Whitney U test with Holm-Bonferroni correction for multiple testing (number of tests = 6) was performed in panel B.

We performed PAS staining on kidney sections of control, WT and SBP-1 KO infected mice to study whether parasite sequestration and parasite load had an influence on the histopathology of the experimental MAKI. Acute tubular injury with vacuolization of tubular epithelial cells and loss of the brush border in proximal tubular epithelial cells was present in both WT and SBP-1 KO infected mice (**Figure 6A**). These observations were more pronounced in the cortex of the kidney. Similar to the *Pb*NK65-E infected mice, glomerulosclerosis was detected with the collapse of some glomerular capillary tufts, in the kidneys of both WT and SBP-1 KO infected mice at day 7 and day 8 p.i. (**Figure 6A** and **Supplementary Figure 3**). Between 1 to 15% of the glomeruli were affected in both infected groups, with no significant difference in the percentage of affected glomeruli between WT and SBP-1 KO infected mice (**Figure 6B**). Parasite sequestration was not detected in glomeruli or tubular blood vessels. We observed intravascular accumulation of leukocytes in the kidneys of WT and SBP-1 KO infected mice (**Figures 7A, B**). Infiltration of leukocytes in the renal interstitium was not detected.

Overall, these data indicate that SBP-1 mediated sequestration and subsequent high parasite load have no major effect in our experimental model of MAKI. Lower BUN values were detected in SBP-1 KO versus WT-infected mice, suggesting pre-renal effects of sequestration-induced high parasite load.

## Discussion

In this study, we describe that MAKI occurs in *Pb*NK65-infected C57BL/6 mice, in parallel with MA-ARDS (Van den Steen et al., 2010). These two complications are most likely independent of each other, as MAKI may also occur in mice without MA-ARDS (Elias et al., 2012). MAKI pathology was characterized by vacuolization in the proximal tubular epithelial cells and collapse of glomerular capillary tufts. The collapse of glomerular capillary tufts in our mouse model has similarities with the collapsing focal segmental glomerulosclerosis (FSGS) described in patients infected with *P. falciparum* (Niang et al., 2008; Kute et al., 2013; Sehar et al., 2015; Amoura et al., 2020). However, such collapse was observed in less than 15% of the glomeruli and the tubular epithelial damage was relatively mild, indicating a less severe and/or earlier form of AKI than collapsing FSGS. The glomerular filtration barrier is composed of fenestrated endothelial cells, the glomerular basement membrane and podocytes (Greka and Mundel, 2012; Wen et al., 2018). FSGS is characterized by damage to podocytes, which damages the glomerular filter barrier and leads to proteinuria. This is in accordance with the occurrence of high proteinuria (>1000 µg/mg of albumin/creatinine) in malaria infected mice with AKI, which is suggestive of glomerular pathology.

The ART + CQ treated mice showed rapid decrease in proteinuria and decrease in histopathological features with a significant decrease in the number of collapsed glomerular tufts. These data indicate the resolution of the glomerular damage or glomerulosclerosis and regeneration of glomerular tissue. Similar to these findings, Remuzzi et al. found in an experimental rat model for progressive kidney disease that after 10 weeks of ACE inhibition more than 20% of glomeruli were completely free of sclerosis, whereas before treatment all glomeruli had some degree of sclerosis (Remuzzi et al., 2006). In contrast, Amoura et al. still observed collapsing FSGS in 91% of kidney biopsies after successful treatment of malaria in patients (Amoura et al., 2020). The difference in resolution between our model and the malaria patients might be due to a difference in severity of glomerulosclerosis. Amoura et al. showed that 64% of the glomeruli from the kidney biopsies displayed segmental and sclerotic lesions, a much higher percentage than what we observed in our model (<15%), which suggests more severe glomerulosclerosis in the studied malaria patients. Furthermore, Wharram et al. shows in genetically engineered rats that a threshold of 20% podocyte loss determines whether repair could occur or whether progressive sclerosis develops (Wharram et al., 2005). Therefore, the percentage of affected podocytes may be lower in our MAKI model compared with the malaria patients in the study of Amoura et al. (Amoura et al., 2020).

Tubular vacuolization has been observed in many renal diseases (Christensen and Maunsbach, 1979; Isik et al., 2006; Kambham et al., 2007; Ding et al., 2020). Vacuolization in the cytoplasm may result from accumulation of a variety of substances, such as lipid droplets, water, glycogen and plasma (Morishita et al., 2005; Dickenmann et al., 2008; Declèves et al., 2014). The precise composition and origin of these vacuoles is not yet clear. However, with the PAS staining, which detects carbohydrates, we noticed that these vacuoles did not contain glycogen. Furthermore, proteinuria may lead to damage to tubular epithelial cells, as documented by Wang et al. (Wang et al., 2018). The AKI pathology in our malaria mouse model is also characterized by significant proteinuria. Therefore, the leaking proteins due to the damaged glomerular filter barrier might lead to damage and vacuolization in the tubular epithelial cells in our MAKI model.

Previously, it has been proposed that parasite sequestration in the kidney may play a role in the pathogenesis of AKI in malaria (Katsoulis et al., 2021). In post-mortem studies, sequestration of *P. falciparum* parasites was higher in glomerular and peritubular capillaries of patients with AKI compared to malaria patients without AKI (Nguansangiam et al., 2007). We did not observe renal sequestration in the WT *Pb*NK65 infected mice. This is in accordance with the results of Franke-Fayard et al., who did not detect major parasite accumulation in perfused kidneys of C57BL/6 mice infected with *Pb*ANKA (Franke-Fayard et al., 2005). This suggests that the kidneys are not a major site of sequestration of *P. berghei* parasites. However, sequestration does occur in other sites, mainly the lungs and umbilical fat tissue and mediate a high parasite load (Franke-Fayard et al., 2005; De Niz et al., 2016; Possemiers et al., 2021). Our data show that the sequestration-mediated high parasite load is dispensable for the occurrence of MAKI.

An important characteristic of AKI pathogenesis is the upregulation of cytokine expression and increased immune cell accumulation and infiltration (Zuk and Bonventre, 2016). In our model, we found a significant increase in the expression of renal IFN-γ and TNF-α. Despite a significant difference in parasite load between WT and SBP-1 KO infected mice, no difference in renal inflammation was observed. We previously observed that parasite sequestration and load had no effect on the induction of lung inflammation in experimental MA-ARDS (Possemiers et al., 2021). In contrast, Plewes et al. observed a significant correlation between parasite load, immune activation and MAKI severity in adult patients with severe falciparum malaria (Plewes et al., 2014).

Surprisingly, BUN levels were significantly different between WT and SBP-1 KO infected mice at day 8 p.i., although proteinuria, renal inflammation and kidney histology were similar in both infected groups. BUN levels are inversely correlated with the decline of kidney function (Seki et al., 2019). However, BUN levels may also be affected by extrarenal factors (Seki et al., 2019). Decreased renal perfusion related to higher parasite load and peripheral sequestration in the WT infected mice may lead to reduction in glomerular filtration rate and increased BUN levels. Moreover, malaria parasites produce high levels of ammonia, which is converted by the liver to urea through the urea cycle (Zeuthen et al., 2006). Therefore, the higher parasite load in the WT infected mice could lead to higher ammonia production by the parasites and may result in increased BUN levels.

The significant transcriptional upregulation of renal HO-1 that we observed in both WT and SBP-1 KO infected mice is important to protect the kidneys from more damage *via* its anti-inflammatory effects. Ramos et al. also found HO-1 mRNA and protein induction in the kidneys of *P. chabaudi* infected C57BL/6 mice and showed that heme catabolism by HO-1 is essential to establish disease tolerance to malaria (Ramos et al., 2019). In contrast, Elias et al. observed a decreased HO-1 expression in kidneys of *Pb*ANKA infected BALB/c mice with AKI (Elias et al., 2012).

Furthermore, we observed that renal KIM-1 mRNA expression was significantly increased in WT and SBP-1 KO infected mice. KIM-1, a transmembrane protein that is expressed in damaged tubular epithelial cells, is widely recognized as an early biomarker of AKI (Bonventre and Yang, 2010; Tian et al., 2017). In accordance with our data, Punsawad et al. detected with immunohistochemical staining a significant increase in KIM-1 expression in proximal tubular cells in all kidney tissues from severe *P. falciparum* malaria patients with AKI (Punsawad and Viriyavejakul, 2017). Various other mouse models of AKI were associated with increased renal KIM-1 expression (Yang et al., 2018; Huang et al., 2020). The renal KIM-1 expression in our malaria mouse model was lower than in cisplatin and LPS induced AKI models. This suggests that the AKI pathology that we observe is less severe and/or at an early stage. This early stage AKI was characterized by intravascular leukocyte accumulation but no leukocyte infiltration in the renal interstitium. Similarly, the majority of patients infected with *P. falciparum* with MAKI showed mainly intravascular localization of leukocytes, only a minority of the patients had a significant mononuclear cell infiltration in the renal interstitium (Nguansangiam et al., 2007). In contrast, Elias *et al.* showed mild mononuclear cell infiltration at day 7 p.i. and pro-inflammatory hypercellularity at day 15 p.i. in the renal interstitium in *Pb*ANKA infected BALB/c mice (Elias et al., 2012).

In conclusion, we describe in this study a model of experimental AKI in malaria with important similarities to AKI in malaria patients. Antimalarial treatment induced the resolution of AKI pathology with significantly decreased proteinuria, tubular injury and collapsing glomerular tufts. Moreover, parasite sequestration and subsequent high parasite load did not affect the AKI pathology.

## Data availability statement

The original contributions presented in the study are included in the article/**Supplementary Material**. Further inquiries can be directed to the corresponding authors.

## Ethics statement

The animal study was reviewed and approved by Animal Ethics Committee KU Leuven.

## Author contributions

HP, EP, FP, and SK performed the experiments. HP analysed the data. PV and HP conceived the study. PK analysed the histological sections. HP and PV wrote the first drafts of the manuscript. All authors critically read and edited the manuscript. All authors read and approved the final manuscript.

## Funding

This work has been supported by the Research Foundation Flanders (F.W.O. Vlaanderen, URL: https://www.fwo.be/) (Grant No G0C9720N to PV) and the Research Fund (C1 project C16/17/010 to PV) of the KU Leuven (URL: https://www.kuleuven.be/kuleuven/). HP holds an aspirant PhD fellowship of the F.W.O. Vlaanderen. EP is a recipient of the L’Oréal-Unesco Women for Sciences FWO PhD fellowship.

## Acknowledgments

The authors thank Prof. B. Sprangers for interesting discussions.

## Conflict of interest

The authors declare that the research was conducted in the absence of any commercial or financial relationships that could be construed as a potential conflict of interest.

## Publisher’s note

All claims expressed in this article are solely those of the authors and do not necessarily represent those of their affiliated organizations, or those of the publisher, the editors and the reviewers. Any product that may be evaluated in this article, or claim that may be made by its manufacturer, is not guaranteed or endorsed by the publisher.

## Supplementary material

Supplementary Figure 1 Antimalarial treatment decreased proteinuria in PbNK65-E infected mice. C57BL/6 mice were infected with PbNK65-E and were treated daily from day 8 until day 12 p.i. with 10 mg/kg ART + 30 mg/kg CQ (ART+CQ). **(A–C)** Albumin, creatinine and albumin/creatinine ratios in the urine were determined. Asterisks above data points indicate significant differences compared to control mice, asterisks above a horizontal line show significant differences between different time points. Data of two experiments, Control: n = 8, untreated *Pb*NK65-E infected mice (UT) d8: n = 8-9, ART+CQ d9-d19: n = 4-9. Mann-Whitney U test with Holm-Bonferroni correction for multiple testing (number of tests = 6) was performed.

Supplementary Figure 2 Antimalarial treatment decreased collapse of glomerular capillary tuft in kidneys of PbNK65-E infected mice. C57BL/6 mice were infected with PbNK65-E parasites and kidneys were dissected at the indicated times. From day 8 p.i., mice were treated daily with antimalarial drugs (ART + CQ). Sections were stained with PAS. Representative images are shown (original magnification x20, bar = 100 µm). Red frames indicate collapse of glomerular tufts. Data of two experiments, Control: n = 6, untreated PbNK65-E infected mice (UT) d8: n = 8, ART+CQ d12: n = 8, ART+CQ d19: n = 6.

Supplementary Figure 3 Similar collapse of glomerular capillary tuft in kidneys of WT and SBP-1 KO infected mice. C57BL/6 mice were infected with WT and SBP-1 KO *Pb*NK65 parasites and at day 7 and 8 p.i. kidneys were dissected. Sections were stained with PAS. Representative images are shown (original magnification x20, bar = 100 µm). Red frames indicate collapse of glomerular tufts. Data of two experiments, Control: n = 6, WT d7-d8: n = 5-6, SBP-1 KO d7-d8: n = 5.
