# Supplementary figures and images for: Experimental malaria-associated acute kidney injury is independent of parasite sequestration and resolves upon antimalarial treatment
Source: Front Cell Infect Microbiol. 2022 Aug 8;12:915792. doi: 10.3389/fcimb.2022.915792 (PMC9394429; doi:10.3389/fcimb.2022.915792)

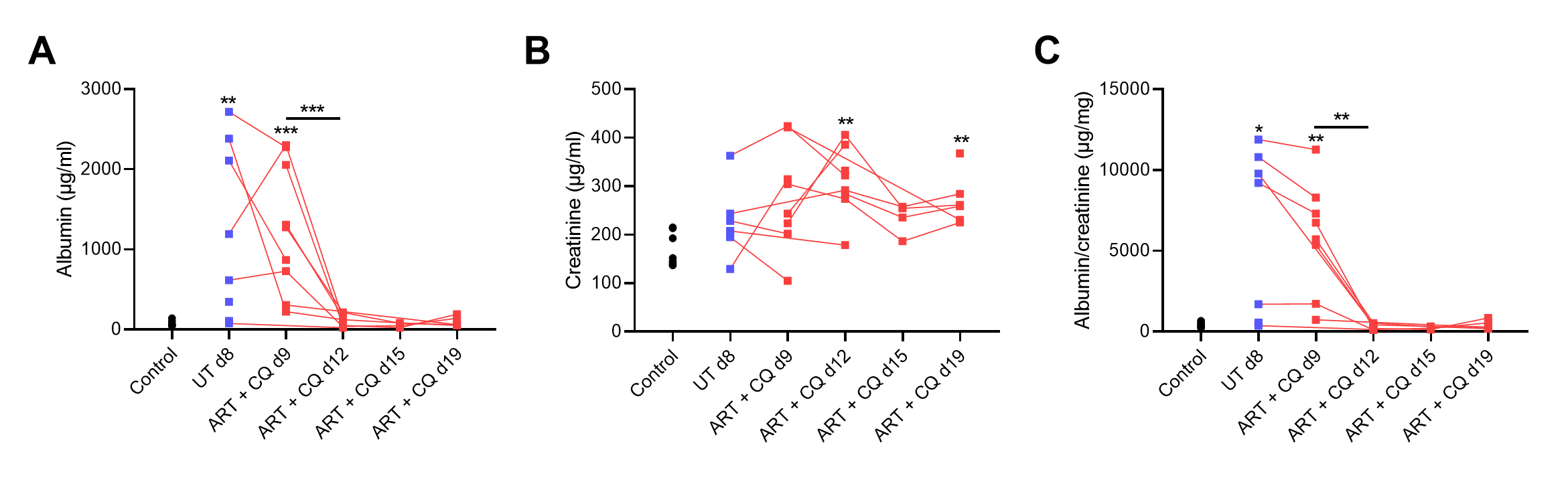

Supplement: Supplementary file 1 — Supplementary Figure 1 Antimalarial treatment decreased proteinuria in PbNK65-E infected mice. C57BL/6 mice were infected with PbNK65-E and were treated daily from day 8 until day 12 p.i. with 10 mg/kg ART + 30 mg/kg CQ (ART+CQ). (A–C) Albumin, creatinine and albumin/creatinine ratios in the urine were determined. Asterisks above data points indicate significant differences compared to control mice, asterisks above a horizontal line show significant differences between different time points. Data of two experiments, Control: n = 8, untreated PbNK65-E infected mice (UT) d8: n = 8-9, ART+CQ d9-d19: n = 4-9. Mann-Whitney U test with Holm-Bonferroni correction for multiple testing (number of tests = 6) was performed. [file Image_1.tif]

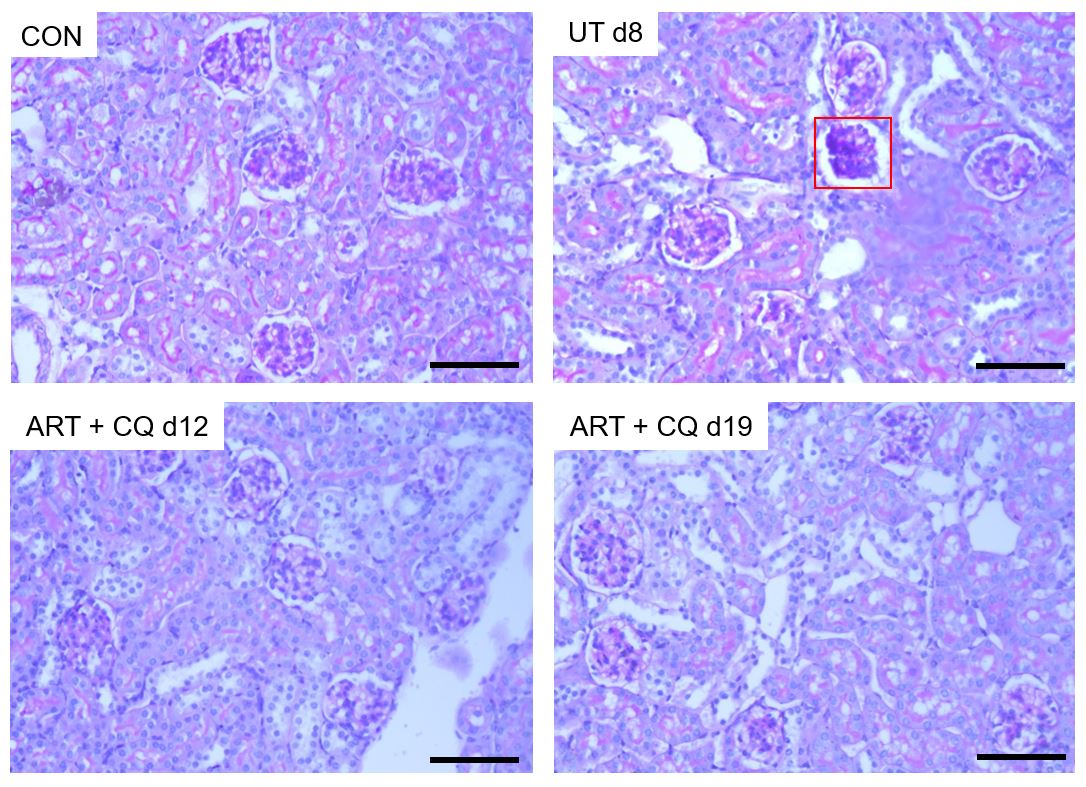

Supplement: Supplementary file 2 — Supplementary Figure 2 Antimalarial treatment decreased collapse of glomerular capillary tuft in kidneys of PbNK65-E infected mice. C57BL/6 mice were infected with PbNK65-E parasites and kidneys were dissected at the indicated times. From day 8 p.i., mice were treated daily with antimalarial drugs (ART + CQ). Sections were stained with PAS. Representative images are shown (original magnification x20, bar = 100 µm). Red frames indicate collapse of glomerular tufts. Data of two experiments, Control: n = 6, untreated PbNK65-E infected mice (UT) d8: n = 8, ART+CQ d12: n = 8, ART+CQ d19: n = 6. [file Image_2.jpeg]

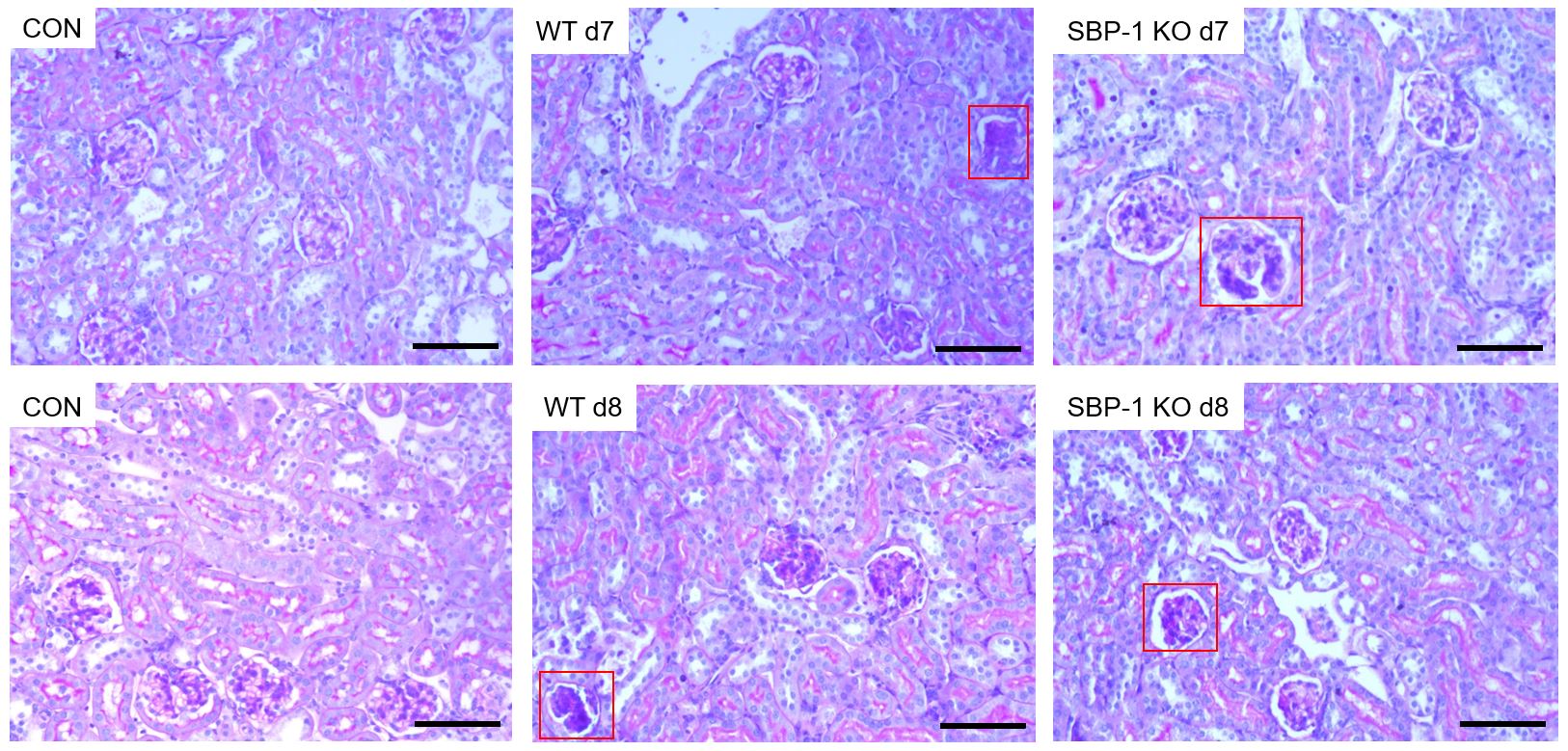

Supplement: Supplementary file 3 — Supplementary Figure 3 Similar collapse of glomerular capillary tuft in kidneys of WT and SBP-1 KO infected mice. C57BL/6 mice were infected with WT and SBP-1 KO PbNK65 parasites and at day 7 and 8 p.i. kidneys were dissected. Sections were stained with PAS. Representative images are shown (original magnification x20, bar = 100 µm). Red frames indicate collapse of glomerular tufts. Data of two experiments, Control: n = 6, WT d7-d8: n = 5-6, SBP-1 KO d7-d8: n = 5. [file Image_3.jpeg]
